# 6-(2-Methyl­phen­yl)-3-(3,4,5-trimethoxy­phen­yl)-1,2,4-triazolo[3,4-*b*][1,3,4]thia­diazole

**DOI:** 10.1107/S160053680802062X

**Published:** 2008-07-12

**Authors:** Haitang Du, Haijun Du, Ying An, Shengnan Li

**Affiliations:** a Department of Chemistry, College of Science, Tianjin University, Tianjin 300072, People’s Republic of China; b Department of Biology and Environment alTechnology, Guiyang College, Guiyang 550005, People’s Republic of China; c School of Chemistry and Environmental Sciences, Guizhou University for Nationalities, Guiyang 550025, People’s Republic of China

## Abstract

In the mol­ecule of the title compound, C_19_H_18_N_4_O_3_S, the planar central heterocylic ring system is oriented with respect to the trimethoxy­phenyl and 2-methyl­phenyl rings at dihedral angles of 4.43 (3) and 4.32 (3)°, respectively. The dihedral angle between the two benzene rings is 7.65 (4)°. In the crystal structure, inter­molecular C—H⋯N hydrogen bonds link the mol­ecules into centrosymmetric *R*_2_^2^(18) dimers. These dimers are connected *via* a C—H⋯π contact between the 2-methyl­phenyl and trimethoxy­phenyl rings, and a π–π contact between the thia­diazole and trimethoxy­phenyl rings [interplanar distance 3.51 Å, dihedral angles 4.17(4)°]. An intramolecular C—H⋯N hydrogen bond is also present.

## Related literature

For general background, see: Karabasanagouda *et al.* (2007 ▶); Mathew *et al.* (2007 ▶). For ring motif details, see: Bernstein *et al.* (1995 ▶).
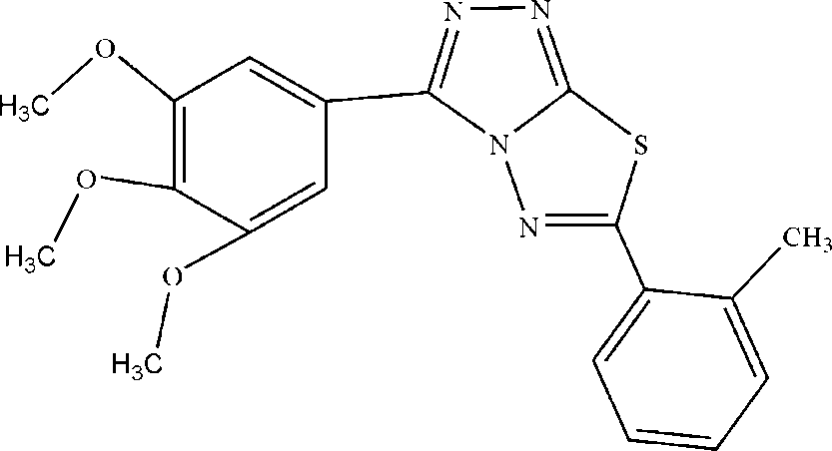


## Experimental

### 

#### Crystal data

C_19_H_18_N_4_O_3_S
*M* *_r_* = 382.43
Monoclinic, 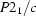

*a* = 7.5108 (15) Å
*b* = 15.950 (3) Å
*c* = 14.096 (3) Å
β = 91.88 (3)°
*V* = 1687.7 (6) Å^3^
*Z* = 4
Mo *K*α radiation
μ = 0.22 mm^−1^
*T* = 113 (2) K
0.24 × 0.20 × 0.16 mm

#### Data collection

Rigaku Saturn CCD area-detector diffractometer
Absorption correction: multi-scan (*CrystalClear*; Rigaku/MSC, 2005 ▶) *T*_min_ = 0.923, *T*_max_ = 0.965
11990 measured reflections
3984 independent reflections
3408 reflections with *I* > 2σ(*I*)
*R*_int_ = 0.031

#### Refinement

*R*[*F* ^2^ > 2σ(*F* ^2^)] = 0.038
*wR*(*F* ^2^) = 0.108
*S* = 1.07
3984 reflections
248 parameters
H-atom parameters constrained
Δρ_max_ = 0.39 e Å^−3^
Δρ_min_ = −0.35 e Å^−3^

### 

Data collection: *CrystalClear* (Rigaku/MSC, 2005 ▶); cell refinement: *CrystalClear*; data reduction: *CrystalStructure* (Rigaku/MSC, 2005 ▶); program(s) used to solve structure: *SHELXS97* (Sheldrick, 2008 ▶); program(s) used to refine structure: *SHELXL97* (Sheldrick, 2008 ▶); molecular graphics: *SHELXTL* (Sheldrick, 2008 ▶) and *PLATON* (Spek, 2003 ▶); software used to prepare material for publication: *SHELXTL* and *PLATON*.

## Supplementary Material

Crystal structure: contains datablocks I, global. DOI: 10.1107/S160053680802062X/hk2487sup1.cif

Structure factors: contains datablocks I. DOI: 10.1107/S160053680802062X/hk2487Isup2.hkl

Additional supplementary materials:  crystallographic information; 3D view; checkCIF report

## Figures and Tables

**Table 1:** Hydrogen-bond geometry (Å, °)

*D*—H⋯*A*	*D*—H	H⋯*A*	*D*⋯*A*	*D*—H⋯*A*
C2—H2⋯N4	0.95	2.45	3.127 (3)	128
C7—H7*C*⋯N2^i^	0.98	2.62	3.524 (3)	154
C19—H19*B*⋯CgA^ii^	0.98	2.90	3.808 (3)	155

Symmetry codes: (i) 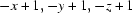; (ii) 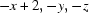. *CgA* is the centroid of the trimethoxyphenyl ring.

**Fig. 1.**
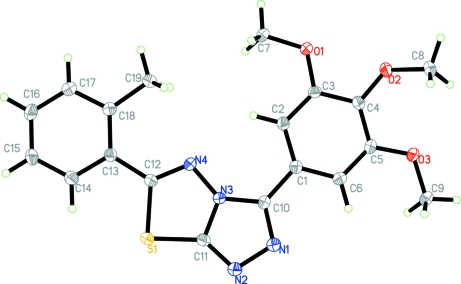
The molecular structure of the title molecule, with the atom-numbering scheme. Displacement ellipsoids are drawn at the 50% probability level.

**Fig. 2.**
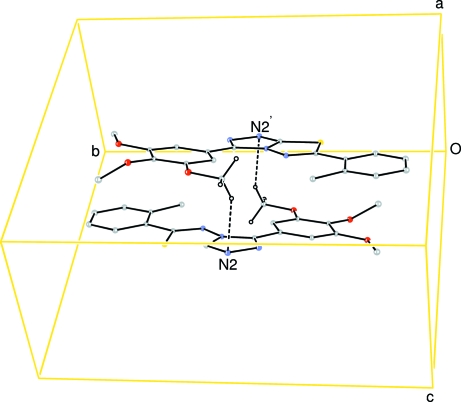
A partial packing diagram of the title compound [symmetry code: (') -x, -y, -z]. Hydrogen bonds are shown as dashed lines.

## supplementary crystallographic information

### Comment

1,2,4-Triazole and 1,3,4-thiadiazole represent one of the most biologically active classes of compounds, possessing a wide spectrum of activities. Various substituted 1,2,4-triazolo[3,4-b]-1,3,4-thiadiazoles are associated with diverse pharmacological activities such as antimicrobial (Karabasanagouda *et al.*,  2007) and anti-inflammatory activity (Mathew *et al.*,  2007). We report herein the crystal structure of the title compound.

In the molecule of the title compound (Fig. 1) the bond lengths and angles are within normal ranges. Rings A (C1-C6), B (N1-N3/C10/C11), C (S1/N3/N4/C11/C12) and D (C13-C18) are, of course, planar, and the dihedral angles between them are A/B = 4.63 (4)°, A/C = 4.08 (3)°, A/D = 7.65 (4)°, B/C = 0.94 (3)°, B/D = 4.59 (4)° and C/D = 4.25 (4)°. So, rings B and C are nearly coplanar. The coplanar ring system is oriented with respect to rings A and D at dihedral angles of 4.43 (3)° and 4.32 (3)°, respectively. The intra- molecular C-H···N hydrogen bond (Table 1) results in the formation of a planar six-membered ring E (N3/N4/C1/C2/C10/H2), in which it is oriented with respect to the other rings at dihedral angles of A/E = 3.24 (4)°, B/E = 1.39 (3)°, C/E = 1.03 (3)° and D/E = 5.25 (4)°. So, rings A, B, C and E are also nearly coplanar.

In the crystal structure, intermolecular weak C-H···N hydrogen bonds (Table 1) link the molecules to form a R^2^_2_(18) ring motif (Fig. 2) (Bernstein *et al.*,  1995), in which they may be effective in the stabilization of the structure. The C—H···π contact (Table 1) between the 2-methylphenyl and trimethoxyphenyl rings and a π—π contact between the thiadiazole and trimethoxyphenyl rings CgC···CgA^i^ [symmetry code: (i) 1 - x, 1 - y, 1 - z] further stabilize the structure, with centroid-centroid distance of 3.506 (1) Å.

### Experimental

For the preparation of the title compound, 4-amino-5-(3,4,5-trimethoxyphenyl) -4H-1,2,4-triazole-3-thiol (0.01 M) and 2-methylbenzoic acid (0.01 M) were dissolved in dry phosphorous oxychloride (10 ml). The resulted solution was further heated under reflux for 7 h. The reaction mixture was cooled to room temperature and the mixture was gradually poured onto crushed ice with stirring. Finally, powdered potassium carbonate and the required amount of solid potassium hydroxide were added until the pH of the mixture was raised to 8, to remove the excess of phosphorous oxychloride. The mixture was allowed to stand overnight and the solid was separated. It was filtered, washed with cold water, and then dried. Crystals suitable for X-ray analysis were obtained by the recrystallization of the solid residue from a mixture of N,N-dimethyl- formamide/ethanol (1:1) by slow evaporation at room temperature.

### Refinement

H atoms were positioned geometrically, with C-H = 0.95 and 0.98 Å for aromatic and methyl H, respectively, and constrained to ride on their parent atoms with U_iso_(H) = xU_eq_(C), where x = 1.5 for methyl H and x = 1.2 for aromatic H atoms.

### Crystal data

**Table:**

C_19_H_18_N_4_O_3_S	*F*_000_ = 800
*M**_r_* = 382.43	*D*_x_ = 1.505 Mg m^−^^3^
Monoclinic, *P*2_1_/*c*	Melting point: 435 K
Hall symbol: -P 2ybc	Mo *K*α radiation λ = 0.71073 Å
*a* = 7.5108 (15) Å	Cell parameters from 4684 reflections
*b* = 15.950 (3) Å	θ = 1.9–27.9º
*c* = 14.096 (3) Å	µ = 0.22 mm^−^^1^
β = 91.88 (3)º	*T* = 113 (2) K
*V* = 1687.7 (6) Å^3^	Prism, colorless
*Z* = 4	0.24 × 0.20 × 0.16 mm

### Data collection

**Table:**

Rigaku Saturn CCD area-detector diffractometer	3984 independent reflections
Radiation source: rotating anode	3408 reflections with *I* > 2σ(*I*)
Monochromator: confocal	*R*_int_ = 0.031
Detector resolution: 7.31 pixels mm^-1^	θ_max_ = 27.9º
*T* = 113(2) K	θ_min_ = 1.9º
ω and φ scans	*h* = −9→9
Absorption correction: multi-scan(CrystalClear; Rigaku/MSC, 2005)	*k* = −20→20
*T*_min_ = 0.923, *T*_max_ = 0.965	*l* = −17→18
11990 measured reflections	

### Refinement

**Table:**

Refinement on *F*^2^	Secondary atom site location: difference Fourier map
Least-squares matrix: full	Hydrogen site location: inferred from neighbouring sites
*R*[*F*^2^ > 2σ(*F*^2^)] = 0.038	H-atom parameters constrained
*wR*(*F*^2^) = 0.108	*w* = 1/[σ^2^(*F*_o_^2^) + (0.0664*P*)^2^ + 0.1888*P*] where *P* = (*F*_o_^2^ + 2*F*_c_^2^)/3
*S* = 1.07	(Δ/σ)_max_ = 0.002
3984 reflections	Δρ_max_ = 0.39 e Å^−^^3^
248 parameters	Δρ_min_ = −0.35 e Å^−^^3^
Primary atom site location: structure-invariant direct methods	Extinction correction: none

### Special details

**Table:**

Geometry. All e.s.d.'s (except the e.s.d. in the dihedral angle between two l.s. planes) are estimated using the full covariance matrix. The cell e.s.d.'s are taken into account individually in the estimation of e.s.d.'s in distances, angles and torsion angles; correlations between e.s.d.'s in cell parameters are only used when they are defined by crystal symmetry. An approximate (isotropic) treatment of cell e.s.d.'s is used for estimating e.s.d.'s involving l.s. planes.
Refinement. Refinement of F^2^ against ALL reflections. The weighted R-factor wR and goodness of fit S are based on F^2^, conventional R-factors R are based on F, with F set to zero for negative F^2^. The threshold expression of F^2^ > 2sigma(F^2^) is used only for calculating R-factors(gt) etc. and is not relevant to the choice of reflections for refinement. R-factors based on F^2^ are statistically about twice as large as those based on F, and R- factors based on ALL data will be even larger.

### Fractional atomic coordinates and isotropic or equivalent isotropic displacement parameters (Å^2^)

**Table:**

	*x*	*y*	*z*	*U*_iso_*/*U*_eq_	
S1	0.45611 (5)	0.68554 (2)	0.69693 (2)	0.02005 (12)	
O1	0.04396 (14)	0.40664 (6)	0.30838 (7)	0.0227 (2)	
O2	0.03955 (14)	0.24866 (6)	0.34593 (7)	0.0223 (2)	
O3	0.17803 (14)	0.18588 (6)	0.51532 (7)	0.0209 (2)	
N1	0.43365 (17)	0.44321 (7)	0.69492 (9)	0.0208 (3)	
N2	0.48819 (18)	0.51377 (8)	0.74703 (9)	0.0226 (3)	
N3	0.36258 (15)	0.55345 (7)	0.61030 (8)	0.0156 (2)	
N4	0.31020 (15)	0.61420 (7)	0.54614 (8)	0.0164 (2)	
C1	0.27851 (18)	0.41101 (8)	0.54193 (10)	0.0162 (3)	
C2	0.20134 (19)	0.44117 (8)	0.45750 (10)	0.0176 (3)	
H2	0.2018	0.4995	0.4439	0.021*	
C3	0.12374 (18)	0.38475 (9)	0.39346 (10)	0.0173 (3)	
C4	0.12199 (18)	0.29857 (8)	0.41238 (10)	0.0170 (3)	
C5	0.19617 (18)	0.26966 (8)	0.49906 (10)	0.0169 (3)	
C6	0.27654 (18)	0.32539 (8)	0.56254 (10)	0.0174 (3)	
H6	0.3303	0.3053	0.6201	0.021*	
C7	0.0480 (2)	0.49299 (9)	0.28278 (11)	0.0232 (3)	
H7A	−0.0099	0.5263	0.3314	0.035*	
H7B	−0.0152	0.5009	0.2215	0.035*	
H7C	0.1720	0.5112	0.2778	0.035*	
C8	0.1222 (2)	0.17048 (9)	0.32363 (12)	0.0276 (4)	
H8A	0.2513	0.1750	0.3349	0.041*	
H8B	0.0968	0.1568	0.2568	0.041*	
H8C	0.0751	0.1262	0.3639	0.041*	
C9	0.2468 (2)	0.15402 (9)	0.60340 (11)	0.0247 (3)	
H9A	0.3756	0.1639	0.6083	0.037*	
H9B	0.2232	0.0937	0.6069	0.037*	
H9C	0.1890	0.1826	0.6557	0.037*	
C10	0.35879 (18)	0.46709 (8)	0.61313 (10)	0.0164 (3)	
C11	0.44294 (18)	0.57788 (9)	0.69380 (10)	0.0182 (3)	
C12	0.35200 (18)	0.68710 (8)	0.58207 (9)	0.0159 (3)	
C13	0.32100 (18)	0.76838 (8)	0.53561 (10)	0.0167 (3)	
C14	0.3639 (2)	0.84107 (9)	0.58780 (11)	0.0212 (3)	
H14	0.4101	0.8357	0.6510	0.025*	
C15	0.3405 (2)	0.92022 (9)	0.54955 (11)	0.0240 (3)	
H15	0.3724	0.9686	0.5855	0.029*	
C16	0.2700 (2)	0.92787 (9)	0.45800 (11)	0.0245 (3)	
H16	0.2521	0.9819	0.4309	0.029*	
C17	0.22563 (19)	0.85687 (9)	0.40588 (11)	0.0210 (3)	
H17	0.1762	0.8634	0.3434	0.025*	
C18	0.25090 (18)	0.77609 (9)	0.44191 (10)	0.0174 (3)	
C19	0.2042 (2)	0.70232 (9)	0.37935 (10)	0.0206 (3)	
H19A	0.1633	0.7224	0.3166	0.031*	
H19B	0.1093	0.6697	0.4079	0.031*	
H19C	0.3096	0.6669	0.3727	0.031*	

### Atomic displacement parameters (Å^2^)

**Table:**

	*U*^11^	*U*^22^	*U*^33^	*U*^12^	*U*^13^	*U*^23^
S1	0.0259 (2)	0.01750 (18)	0.01647 (19)	−0.00076 (13)	−0.00384 (14)	−0.00149 (12)
O1	0.0347 (6)	0.0148 (5)	0.0181 (5)	0.0021 (4)	−0.0071 (4)	0.0011 (4)
O2	0.0258 (6)	0.0165 (5)	0.0242 (6)	0.0014 (4)	−0.0056 (4)	−0.0036 (4)
O3	0.0278 (6)	0.0142 (5)	0.0203 (5)	−0.0019 (4)	−0.0035 (4)	0.0032 (4)
N1	0.0244 (7)	0.0189 (6)	0.0190 (6)	0.0012 (5)	−0.0023 (5)	−0.0002 (5)
N2	0.0287 (7)	0.0196 (6)	0.0193 (6)	0.0012 (5)	−0.0038 (5)	−0.0006 (5)
N3	0.0173 (6)	0.0158 (5)	0.0136 (5)	0.0000 (4)	−0.0010 (4)	0.0005 (4)
N4	0.0182 (6)	0.0149 (5)	0.0160 (6)	0.0005 (4)	−0.0002 (4)	0.0024 (4)
C1	0.0154 (6)	0.0170 (6)	0.0164 (7)	0.0007 (5)	0.0026 (5)	−0.0006 (5)
C2	0.0202 (7)	0.0150 (6)	0.0177 (7)	0.0015 (5)	0.0012 (5)	−0.0004 (5)
C3	0.0200 (7)	0.0179 (7)	0.0140 (6)	0.0031 (5)	0.0005 (5)	0.0003 (5)
C4	0.0171 (7)	0.0155 (6)	0.0184 (7)	−0.0001 (5)	0.0003 (5)	−0.0022 (5)
C5	0.0167 (7)	0.0142 (6)	0.0199 (7)	0.0010 (5)	0.0034 (5)	0.0014 (5)
C6	0.0168 (7)	0.0190 (7)	0.0165 (7)	0.0019 (5)	0.0005 (5)	0.0020 (5)
C7	0.0316 (8)	0.0161 (7)	0.0217 (7)	0.0028 (6)	−0.0042 (6)	0.0037 (5)
C8	0.0387 (9)	0.0170 (7)	0.0265 (8)	0.0034 (6)	−0.0065 (7)	−0.0061 (6)
C9	0.0335 (9)	0.0177 (7)	0.0227 (8)	−0.0002 (6)	−0.0037 (6)	0.0058 (6)
C10	0.0169 (7)	0.0146 (6)	0.0177 (7)	0.0008 (5)	0.0016 (5)	0.0014 (5)
C11	0.0196 (7)	0.0196 (6)	0.0154 (7)	−0.0006 (5)	−0.0010 (5)	−0.0013 (5)
C12	0.0147 (6)	0.0180 (7)	0.0149 (7)	−0.0003 (5)	0.0002 (5)	−0.0013 (5)
C13	0.0161 (7)	0.0163 (6)	0.0176 (7)	0.0005 (5)	0.0006 (5)	0.0000 (5)
C14	0.0233 (7)	0.0198 (7)	0.0204 (7)	0.0001 (6)	−0.0018 (6)	−0.0023 (6)
C15	0.0287 (8)	0.0161 (6)	0.0271 (8)	−0.0007 (6)	−0.0012 (6)	−0.0030 (6)
C16	0.0297 (8)	0.0168 (7)	0.0270 (8)	0.0013 (6)	0.0005 (6)	0.0025 (6)
C17	0.0218 (7)	0.0216 (7)	0.0198 (7)	0.0011 (6)	0.0004 (5)	0.0019 (5)
C18	0.0163 (7)	0.0190 (7)	0.0169 (7)	0.0004 (5)	0.0015 (5)	0.0003 (5)
C19	0.0245 (8)	0.0200 (7)	0.0172 (7)	−0.0012 (6)	−0.0015 (5)	−0.0009 (5)

### Geometric parameters (Å, °)

**Table:**

S1—C11	1.7205 (15)	C7—H7A	0.9800
S1—C12	1.7749 (15)	C7—H7B	0.9800
O1—C3	1.3682 (17)	C7—H7C	0.9800
O1—C7	1.4244 (16)	C8—H8A	0.9800
O2—C4	1.3625 (16)	C8—H8B	0.9800
O2—C8	1.4325 (17)	C8—H8C	0.9800
O3—C5	1.3633 (16)	C9—H9A	0.9800
O3—C9	1.4229 (17)	C9—H9B	0.9800
N1—C10	1.3221 (18)	C9—H9C	0.9800
N1—N2	1.3978 (17)	C12—C13	1.4677 (19)
N2—C11	1.3067 (18)	C13—C14	1.4048 (19)
N3—C11	1.3619 (18)	C13—C18	1.411 (2)
N3—N4	1.3742 (16)	C14—C15	1.382 (2)
N3—C10	1.3783 (17)	C14—H14	0.9500
N4—C12	1.3026 (17)	C15—C16	1.384 (2)
C1—C2	1.392 (2)	C15—H15	0.9500
C1—C6	1.3964 (19)	C16—C17	1.385 (2)
C1—C10	1.4600 (19)	C16—H16	0.9500
C2—C3	1.3893 (19)	C17—C18	1.3956 (19)
C2—H2	0.9500	C17—H17	0.9500
C3—C4	1.4003 (19)	C18—C19	1.5050 (19)
C4—C5	1.404 (2)	C19—H19A	0.9800
C5—C6	1.3855 (19)	C19—H19B	0.9800
C6—H6	0.9500	C19—H19C	0.9800
			
C11—S1—C12	88.13 (6)	O3—C9—H9A	109.5
C3—O1—C7	117.15 (11)	O3—C9—H9B	109.5
C4—O2—C8	117.97 (11)	H9A—C9—H9B	109.5
C5—O3—C9	117.47 (11)	O3—C9—H9C	109.5
C10—N1—N2	109.59 (11)	H9A—C9—H9C	109.5
C11—N2—N1	105.19 (12)	H9B—C9—H9C	109.5
C11—N3—N4	118.44 (11)	N1—C10—N3	107.73 (12)
C11—N3—C10	105.66 (11)	N1—C10—C1	125.26 (12)
N4—N3—C10	135.89 (12)	N3—C10—C1	126.93 (12)
C12—N4—N3	108.26 (11)	N2—C11—N3	111.83 (13)
C2—C1—C6	120.59 (13)	N2—C11—S1	138.82 (11)
C2—C1—C10	121.79 (12)	N3—C11—S1	109.35 (10)
C6—C1—C10	117.59 (12)	N4—C12—C13	125.61 (13)
C3—C2—C1	119.05 (13)	N4—C12—S1	115.82 (10)
C3—C2—H2	120.5	C13—C12—S1	118.57 (10)
C1—C2—H2	120.5	C14—C13—C18	119.37 (13)
O1—C3—C2	124.52 (12)	C14—C13—C12	117.71 (13)
O1—C3—C4	114.26 (12)	C18—C13—C12	122.93 (12)
C2—C3—C4	121.23 (13)	C15—C14—C13	121.71 (14)
O2—C4—C3	116.72 (12)	C15—C14—H14	119.1
O2—C4—C5	124.38 (12)	C13—C14—H14	119.1
C3—C4—C5	118.83 (12)	C14—C15—C16	118.96 (14)
O3—C5—C6	124.34 (13)	C14—C15—H15	120.5
O3—C5—C4	115.42 (12)	C16—C15—H15	120.5
C6—C5—C4	120.22 (12)	C15—C16—C17	120.04 (14)
C5—C6—C1	120.03 (13)	C15—C16—H16	120.0
C5—C6—H6	120.0	C17—C16—H16	120.0
C1—C6—H6	120.0	C16—C17—C18	122.30 (14)
O1—C7—H7A	109.5	C16—C17—H17	118.8
O1—C7—H7B	109.5	C18—C17—H17	118.8
H7A—C7—H7B	109.5	C17—C18—C13	117.60 (13)
O1—C7—H7C	109.5	C17—C18—C19	118.83 (13)
H7A—C7—H7C	109.5	C13—C18—C19	123.57 (12)
H7B—C7—H7C	109.5	C18—C19—H19A	109.5
O2—C8—H8A	109.5	C18—C19—H19B	109.5
O2—C8—H8B	109.5	H19A—C19—H19B	109.5
H8A—C8—H8B	109.5	C18—C19—H19C	109.5
O2—C8—H8C	109.5	H19A—C19—H19C	109.5
H8A—C8—H8C	109.5	H19B—C19—H19C	109.5
H8B—C8—H8C	109.5		
			
C10—N1—N2—C11	0.13 (16)	C2—C1—C10—N1	179.68 (13)
C11—N3—N4—C12	−0.04 (16)	C6—C1—C10—N1	1.6 (2)
C10—N3—N4—C12	−178.51 (15)	C2—C1—C10—N3	3.3 (2)
C6—C1—C2—C3	−0.6 (2)	C6—C1—C10—N3	−174.73 (13)
C10—C1—C2—C3	−178.64 (13)	N1—N2—C11—N3	−0.07 (16)
C7—O1—C3—C2	2.7 (2)	N1—N2—C11—S1	−179.34 (13)
C7—O1—C3—C4	−177.29 (12)	N4—N3—C11—N2	−178.90 (12)
C1—C2—C3—O1	−179.99 (13)	C10—N3—C11—N2	−0.01 (16)
C1—C2—C3—C4	0.0 (2)	N4—N3—C11—S1	0.58 (15)
C8—O2—C4—C3	138.95 (14)	C10—N3—C11—S1	179.48 (9)
C8—O2—C4—C5	−44.2 (2)	C12—S1—C11—N2	178.60 (18)
O1—C3—C4—O2	−1.28 (18)	C12—S1—C11—N3	−0.68 (10)
C2—C3—C4—O2	178.74 (12)	N3—N4—C12—C13	179.15 (12)
O1—C3—C4—C5	−178.28 (12)	N3—N4—C12—S1	−0.53 (14)
C2—C3—C4—C5	1.7 (2)	C11—S1—C12—N4	0.74 (11)
C9—O3—C5—C6	−0.1 (2)	C11—S1—C12—C13	−178.97 (11)
C9—O3—C5—C4	−178.32 (12)	N4—C12—C13—C14	175.74 (13)
O2—C4—C5—O3	−1.3 (2)	S1—C12—C13—C14	−4.59 (17)
C3—C4—C5—O3	175.43 (12)	N4—C12—C13—C18	−4.3 (2)
O2—C4—C5—C6	−179.60 (13)	S1—C12—C13—C18	175.39 (11)
C3—C4—C5—C6	−2.9 (2)	C18—C13—C14—C15	−0.5 (2)
O3—C5—C6—C1	−175.88 (13)	C12—C13—C14—C15	179.46 (13)
C4—C5—C6—C1	2.2 (2)	C13—C14—C15—C16	1.2 (2)
C2—C1—C6—C5	−0.5 (2)	C14—C15—C16—C17	−0.6 (2)
C10—C1—C6—C5	177.61 (12)	C15—C16—C17—C18	−0.7 (2)
N2—N1—C10—N3	−0.13 (15)	C16—C17—C18—C13	1.3 (2)
N2—N1—C10—C1	−177.07 (12)	C16—C17—C18—C19	−178.14 (14)
C11—N3—C10—N1	0.09 (15)	C14—C13—C18—C17	−0.7 (2)
N4—N3—C10—N1	178.69 (14)	C12—C13—C18—C17	179.32 (13)
C11—N3—C10—C1	176.96 (13)	C14—C13—C18—C19	178.70 (13)
N4—N3—C10—C1	−4.4 (3)	C12—C13—C18—C19	−1.3 (2)

### Hydrogen-bond geometry (Å, °)

**Table:**

*D*—H···*A*	*D*—H	H···*A*	*D*···*A*	*D*—H···*A*
C2—H2···N4	0.95	2.45	3.127 (3)	128
C7—H7C···N2^i^	0.98	2.62	3.524 (3)	154
C19—H19B···CgA^ii^	0.98	2.90	3.808 (3)	155

Symmetry codes: (i) −*x*+1, −*y*+1, −*z*+1; (ii) −*x*+2, −*y*, −*z*.

## References

[bb1] Bernstein, J., Davies, R. E., Shimoni, L. & Chang, N. L. (1995). *Angew. Chem. Int. Ed. Engl.* **34**, 1555–1573.

[bb2] Karabasanagouda, T., Adhikari, A. V. & Shetty, S. N. (2007). *Eur. J. Med. Chem.* **42**, 521–529. 10.1016/j.ejmech.2006.10.010 17156898

[bb3] Mathew, V., Keshavayya, J., Vaidya, V. P. & Giles, D. (2007). *Eur. J. Med. Chem.* **42**, 823–840. 10.1016/j.ejmech.2006.12.010 17331622

[bb5] Rigaku/MSC. (2005). *CrystalClear* and *CrystalStructure* Rigaku/MSC, The Woodlands, Texas, USA.

[bb6] Sheldrick, G. M. (2008). *Acta Cryst.* A**64**, 112–122. 10.1107/S0108767307043930 18156677

[bb7] Spek, A. L. (2003). *J. Appl. Cryst.* **36**, 7–13.

